# Mitochondrial dysfunction on *Leishmania (Leishmania) amazonensis* induced by ketoconazole: insights into drug mode of action

**DOI:** 10.1590/0074-02760210157

**Published:** 2022-04-29

**Authors:** Débora Cristina de Oliveira Silva Nunes, Mônica Soares Costa, Luiz Borges Bispo-da-Silva, Eloísa Amália Vieira Ferro, Mariana Alves Pereira Zóia, Luiz Ricardo Goulart, Renata Santos Rodrigues, Veridiana de Melo Rodrigues, Kelly Aparecida Geraldo Yoneyama

**Affiliations:** 1 Universidade Federal de Uberlândia, Instituto de Biotecnologia, Laboratório de Bioquímica e Toxinas Animais, Uberlândia, MG, Brasil; 2 Universidade Federal de Uberlândia, Instituto de Ciências Biomédicas, Departamento de Farmacologia, Uberlândia, MG, Brasil; 3 Universidade Federal de Uberlândia, Instituto de Ciências Biomédicas, Laboratório de Imunofisiologia da Reprodução, Uberlândia, MG, Brasil; 4 Universidade Federal de Uberlândia, Instituto de Biotecnologia, Laboratório de Nanobiotecnologia, Uberlândia, MG, Brasil

**Keywords:** cutaneous leishmaniasis, ergosterol, mitochondrial damage, sterol biosynthesis inhibitor

## Abstract

**BACKGROUND:**

*Leishmania* parasites cause leishmaniasis that range from self-limiting cutaneous lesions to more serious forms of the disease. The search for potential drug targets focusing on biochemical and metabolic pathways revealed the sterol biosynthesis inhibitors (SBIs) as a promising approach. In this class of inhibitors is found ketoconazole, a classical inhibitor of 14α-methysterol 14-demethylase.

**OBJECTIVE:**

The present study aimed to better understand the biological response of *Leishmania (Leishmania) amazonensis* promastigotes at the cellular level after ketoconazole treatment.

**METHODS:**

Herein, techniques, such as fluorimetry, flow cytometry, fluorescence microscopy, electron and scanning microscopy were used to investigate the cellular structures and to identify organelles affected by ketoconazole treatment.

**FINDINGS:**

The study demonstrated, for the first time, the effect of ketoconazole on mitochondrion functioning and its probable relationship to cell cycle and death on *L. (L.) amazonensis* promastigotes (IFLA/BR/67/PH8 strain).

**MAIN CONCLUSIONS:**

Ketoconazole-induced mitochondrial damages led to hyperpolarisation of this single organelle and autophagic vacuoles formation, as a parasite survival strategy. These damages did not reflect directly on the parasite cell cycle, but drove the parasites to death, making them susceptible to ketoconazole treatment in *in vitro* models.

Leishmaniasis are caused by *Leishmania* spp. and comprise a spectrum of diseases that vary from self-limiting cutaneous lesions to more serious conditions.^1^ The *Leishmania (Leishmania) amazonensis* species has clinical and epidemiological importance as etiological agent of cutaneous leishmaniasis in Latin America, especially in Brazil and Colombia.^2^ ^,^ ^3^ ^,^ ^4^ *L. (L.) amazonensis* exhibits a broad spectrum of clinical diseases, ranging from cutaneous, cutaneous diffuse, mucocutaneous and visceral.^5^ ^,^ ^6^ It is the main etiological agent of disseminated cutaneous leishmaniasis and, more recently, visceral leishmaniasis in both humans and domestic animals.^7^ ^-^ ^15^ Recent findings indicate that *L. (L.) amazonensis* is increasing its geographical distribution in Brazil, accounting for unusual clinical presentations in new transmission areas.^16^

Currently available treatments for leishmaniasis present contraindications, high toxicity and cost, difficulties in healing, and have also been related to the development of parasite resistance.^1^ ^,^ ^17^ ^,^ ^18^ These problems could be solved, at least partially, by the searching of specific inhibitors of biochemical and metabolic targets that are essential for the parasite survival, with minimal or no effects on the host organisms.

Unlike plasma membranes of animals, which contain cholesterol as the major sterol, certain protozoa as trypanosomatids, yeast, and fungi present ergosterol, that plays a role in regulating membrane fluidity and permeability,^19^ ^,^ ^20^ in addition to serving as precursors for biologically active molecules that regulate growth and development processes.^20^ In *Leishmania*, it was shown that the removal of membrane ergosterol by using a chelating agent, resulted in disruption of lipid platforms, also known as lipid microdomains. These specialised regions of the membrane are enriched with molecules essential for the process of invasion and infection in the host cell. Thus, the disruption of these platforms resulted in reduction of the parasite infectivity index, indicating the importance of ergosterol for the maintenance of such lipid structures, as well as for the biological action of molecules involved in the leishmaniasis pathogenesis.^21^ Synthesis of sterols requires removal of the 14α-methyl groups from sterol precursors. This reaction is catalysed by a microsomal cytochrome P_450_, the sterol 14α-methysterol 14-demethylase (CYP51).^19^ In this regard, the sterol biosynthetic pathway appears as a potential drug target and the sterol biosynthesis inhibitors (SBIs) certainly represent a promising approach.

The SBIs evaluated on trypanosomatids are classified as the allylamines that inhibit squalene-2,3-epoxidase; the azasterols that block the C-24 alkylation reaction; and the imidazole and triazole compounds that inhibit 14α-methylsterol 14-demethylase.^19^ Among SBIs the ketoconazole, commonly used as antifungal, has been shown to produce minimal side effects on vertebrate hosts. More interestingly, clinical studies showed the effectiveness of ketoconazole in leishmaniasis^22^ ^-^ ^27^ and a study showed that ruthenium-ketoconazole complexes (Ru-KTZ) increased the activity toward promastigotes and intracellular amastigotes of *L. major*, did not present toxicity to human or murine normal cells and increased the selectivity toward *Leishmania* parasites, in relation normal human cells, when compared with uncomplexed ketoconazole, or with similar ruthenium compounds not containing ketoconazole.^28^ However, despite leishmaniasis being treated by non-*Leishmania* drugs, such as ketoconazole, the exact mechanism of action of these drugs on *Leishmania* spp. is not clearly understood.

Therefore, the present study aimed to examine and better understand the biological response of *L. (L.) amazonensis* promastigotes at cellular level to the classical SBI ketoconazole. For the first time, the action of the ketoconazole on mitochondrion, autophagic vacuoles, cell death pathway, and cell cycle of *L. (L.) amazonensis* parasites has been demonstrated. We found that ketoconazole is a potent inhibitor of *L. (L.) amazonensis* growth, interferes with parasite viability but is not toxic for host cell, causes multiple alterations in the ultrastructure of promastigotes, affects the structure and function of the mitochondrion, induces autophagic vacuoles formation. Moreover, the drug does not alter the parasite cell cycle, but tends to induce cell death by apoptosis leading to intramacrophage parasites susceptibility to drug.

## MATERIALS AND METHODS

*Chemicals* - Ketoconazole, dimethylsulfoxide (DMSO), penicillin, streptomycin, triton x-100, rhodamine 123, monodansylcadaverine (MDC), RPMI 1640 medium from Sigma Chemical Co. (USA), annexin V FITC apoptosis detection kit from BD Pharmingen, MitoTracker^R^ RedCMXRos from Invitrogen (USA), foetal bovine serum (FBS) from Cultilab (Brazil), and Schneider’s insect medium from LGC Biotecnologia (Brazil) were used in this study. Ketoconazole was dissolved in a 1 M stock of DMSO and stored at -20ºC. During assays new dilutions were carried out to ensure that the DMSO concentration in culture medium did not exceed 0.1%.

*Animals* - Male BALB/c mice (six-eight weeks old) had access to water and standard chow ad libitum. They were kept in a room with controlled temperature (25ºC) and luminosity (12 h light/dark cycles). The experimental procedures were analysed and approved by the Animal Ethics Committee of the Federal University of Uberlândia (CEUA/UFU) under the number 36/2013.

*Cells culture* - Promastigotes of *L. (L.) amazonensis* (IFLA/BR/67/PH8 strain) were cultured in Schneider’s insect medium pH 7.4 containing 10% heat-inactivated FBS, 1% penicillin (100 UI x mL^−1^), and streptomycin (100 µg x mL^−1^). Parasites were kept in a BOD chamber at 23 ± 0.5ºC.

Murine macrophage cell line RAW264.7 (Rio de Janeiro Bank Cell) was cultured in RPMI 1640 medium pH 7.4 supplemented with 5% FBS, penicillin (100 UI x mL^−1^), streptomycin (100 μg x mL^−1^) in 75-cm^2^ flasks. All cell cultures were done at 37ºC in humidified air with 5% CO_2_.

Free amastigotes were obtained from footpad of BALB/c mice previously infected with promastigote forms (1 × 10^7^ cells/footpad) for five to six weeks.^29^ These parasites were cultured in complete Schneider’s insect medium, at 32 ± 0.5ºC pH 5, ensuring that these parasites remained as axenic amastigote forms.^30^

*Antiproliferative and viability assays* - The effect of ketoconazole on cellular proliferation was evaluated. Promastigotes (5 × 10^6^ cells x mL^−1^) were cultured in 25 cm^2^ cell culture flasks containing medium with different drug concentrations (300 to 0.001 μM). After 24, 48, 72, 96 and 120 h of incubation with drug, the parasite concentrations were determined by blind counting using a Neubauer chamber. Three independent experiments were performed, each one in triplicate. The EC_50_ values with 95% confidence limits were calculated by GraphPad Prism 5.0 (GraphPad Software Inc., San Diego, USA).

Viability assay was carried out on promastigote forms using the MTT (3-(4,5-dimethylthiazol-2-yl)-2,5-diphenyl tetrazolium bromide) reagent. Parasites (5 × 10^6^ cells x mL^−1^) were incubated in the absence (viability control) or presence of ketoconazole (200 to 0.097 µM) for 24, 48, and 72 h into 96 wells plate. The internal controls of the experiment were: culture medium only, DMSO only, parasites cultured in the presence of Triton X-100 (death control) and parasites cultured in the presence of culture medium (viability control) (data not shown to make the figure clean). Three independent experiments were performed, all in triplicate. The effective concentration of drug able to cause 50% of citotoxicity (EC_50_) was graphically determined by non-linear regression log in each independent experiment using the Graphpad Prism 5 software (United States); for this, all treatments and the viability control had their absorbances deducted from the absorbance value of the culture medium. Finally, the absorbance of the viability control deducted from the absorbance of the culture medium was used as reference parameter (100% of viability) and the percentages of treatments were calculated from then on.

Moreover, another viability assay was performed: Trypan Blue exclusion test. Parasites (5 x 10^6^ cells x mL^−1^) were cultured in medium for 48 h. Subsequently, the cells were treated with 10 µM ketoconazole (2xEC_50_ of 72 h growth curve) and the number of promastigotes was determined by blind counting using a Neubauer chamber for three consecutive days (72 h) and Trypan blue stain. Three independent experiments were performed, all in triplicate.

*Morphological and ultrastructural analysis* - For evaluating ultrastructural changes, transmission electron (TEM) and scanning electron (SEM) microscopies were performed. For TEM, promastigotes (5 x 10^6^ cells x mL^−1^, log phase) were incubated with or without (control) ketoconazole 10 μM for 72 h. After being treated, the parasites were fixed at 4ºC with 2.5% glutaraldehyde in 0.1 M phosphate buffered saline (PBS) pH 7.2 for 24 h, washed in PBS, post-fixed in PBS containing 0.8% potassium ferrocyanide and 1% osmium tetroxide (OsO_4_) for 1 h and washed once again. Subsequently, the parasites were dehydrated in a gradual series of acetone and set in resin. Ultrathin sections were obtained, contrasted with uranyl acetate and lead citrate and analysed using a Zeiss EM 109 transmission electron microscope (Zeiss, Oberkochen, Germany). For SEM, promastigotes treated or not with ketoconazole 10 μM for 72 h were dehydrated in ethanol, critical point-dried in CO_2_, mounted in stubs, sputtered with a thin gold layer, and analysed using a scanning electron microscope (Zeiss EVO MA10).

*Evaluation of mitochondrial damage* - The fluorescent stain Rhodamine 123 was employed to evaluate the mitochondrial transmembrane potential (∆Ψ_*m*_ ) of promastigotes. Initially, parasites (5 x 10^6^ cells x mL^−1^) were treated with 10 µM ketoconazole or not (control) for 24, 48, and 72 h. Aliquots were incubated with Rhodamine 123 (15 µg x mL^−1^) for 15 min at room temperature and protected from light (dark). Subsequently, the parasites were washed twice with PBS. The cell population analysis was carried out in a flow cytometer (BD Accuri C6 - Biosciences, CA, USA) and 10,000 events were obtained from the region corresponding to the parasites. Three independent experiments were performed.

Furthermore, promastigotes (5 x 10^6^ cells x mL^−1^) treated or not (control) with ketoconazole (10 µM, 72 h) were incubated with MitoTracker^R^ RedCMXRos (300 nM) for 30 min in the dark. Subsequently, parasites were washed with PBS, fixed in paraformaldehyde 1% in cacodylate buffer, and washed twice with PBS. The cell population analysis was performed with a confocal fluorescence microscopy (Zeiss LSM510 Meta).

*Detection of autophagic vacuoles* - The monodansylcadaverine (MDC) labeling was carried out to evaluate the autophagic vacuoles formation. Promastigotes (5 x 10^6^ cells x mL^−1^, log phase) were cultured in absence (control) or presence of ketoconazole 10 μM for 24, 48, and 72 h. Subsequently, 100 µM MDC was added to parasites and incubated for 2 h in the dark. Afterwards, the parasites were washed with PBS, fixed in 1% paraformaldehyde in cacodylate buffer, washed twice with PBS, mounted on microscope slides, and analysed by fluorescence microscopy (excitation wavelength 358 nm and emission wavelength 463 nm). Two independent experiments were performed and the fluorescence intensity was quantified by the software Image J version 1.48.

*Cell cycle analysis* - Initially, the interference of ketoconazole on the cell cycle was evaluated by counting the number of nucleous, kinetoplast and flagella by parasite. Promastigotes (5 x 10^6^ cells x mL^-1^) treated or not (control) with ketoconazole (10 uM, 72 h) labelled with DAPI (1:500, for 1 h) were analysed using a confocal fluorescence microscope (Zeiss LSM510 Meta). For a more detailed study, parasites (5 x 10^6^ cells x mL^−1^, log phase) were incubated for 72 h with or without (control) ketoconazole (10 μM, 72 h) in medium containing 10% FBS. After incubation, parasites were washed and resuspended in ice-cold 70% ethanol in PBS and fixed for 18 h at 4ºC. Subsequently, the parasites were incubated with PBS containing 10 µg x mL^−1^ propidium iodide and 100 µg x mL^−1^ RNAse A for 45 min at 37ºC, protected from light. The cell population was analysed by flow cytometer (BD Accuri C6 - Biosciences, CA, USA) and 10,000 events were obtained from the region corresponding to the parasites. For each phase of the cell cycle, a respective percentage was obtained using the FlowJov10.0.7 software.

*Cell death analysis* - Promastigotes (5 x 10^6^ cells x mL^−1^) were incubated for 72 h with or without (negative control) ketoconazole (10 μM, 72 h); while parasites incubated with formaldehyde 4% for 15 min were considered positive control. Subsequently, a binding buffer containing annexin V-FITC and 2 μg x mL^−1^ propidium iodide (BD Bioscience, Brazil) was added to the parasites and incubated for 15 min at 25ºC in the dark, according to the manufacturer’s instructions. Then, the parasites were washed and the cell population was analysed by flow cytometer (BD Accuri C6 - Biosciences, CA, USA). Thirty thousand events were obtained from the region corresponding to the parasites. The percentages of apoptotic cells were determined using the FlowJo v10.0.7 software.

For Propidium Iodide (PI) and Annexin V (AV) labeling, promastigotes (5 x 10^6^ cells x mL^−1^) treated with ketoconazole (10 μM, 72 h), medium (negative control) or formaldehyde 4% (positive control) were incubated in presence of PI (2 µg x mL^-1^), AV (1 µg x mL^-1^) or both for 15 min at 25ºC, protected from the light. Subsequently, parasites were fixed with 1% paraformaldehyde in a cacodylate buffer, washed and placed on glass coverslips. Finally, the parasites were analysed using a confocal fluorescence microscope (Zeiss LSM510 Meta).

*Intracellular amastigote assay* - Murine macrophage cell line RAW264.7 (4 × 10^5^/well) cultured in RPMI 1640 medium, supplemented with 5% FBS, penicillin (100 UI x mL^−1^), streptomycin (100 μg x mL^−1^) was placed in 24-well plates containing 13-mm diameter glass coverslips and infected with *L. (L.) amazonensis* free amastigotes isolated just before their use (two free amastigotes: 1 macrophage). After 1 h 30, plates were washed with PBS to remove non-internalised amastigotes and RPMI medium alone (control) or containing drugs (200 to 12,5 µM for ketoconazole, double serial dilutions) was added. Experiments were conducted at 37ºC in a 5% CO_2_ humidified incubator for 48 h. Cells on coverslips were fixed and stained with Giemsa stain modified solution for evaluation of the infectivity index; 100 cells per coverslip were blind counted and the infectivity index was determined by multiplying the percentage of macrophages that had phagocytosed at least one parasite by the parasite average per infected macrophage. This assay was carried out in quadruplicate and two independent experiments were performed.

It is worth highlighting the cytotoxic effect of ketoconazole on murine macrophages cell line RAW264.7 was, previously, performed by MTT test at the same drug concentrations used to assess the viability of promastigotes (section 2.4), but within 48 h. Since the macrophages were infected with amastigotes isolated from animal paw just before use and it was not necessary time to promastigote-amastigote differentiation, the infectivity assay was conducted at 48 h, time that allowed better visualisation and counting of the intramacrophage amastigotes.

*Statistics* - Statistical analysis was conducted using GraphPad Prism 5. Each set of results was firstly checked for normal distribution using KolmogorovSmirnov, D’Agostinho and Pearson, and ShapiroWalk tests. Normally distributed data were analysed through one-way-ANOVA followed by Tukey’s post-test or Student test T. Statistically significant differences were assumed when at least p < 0.05.

## RESULTS

**Fig. 1:**
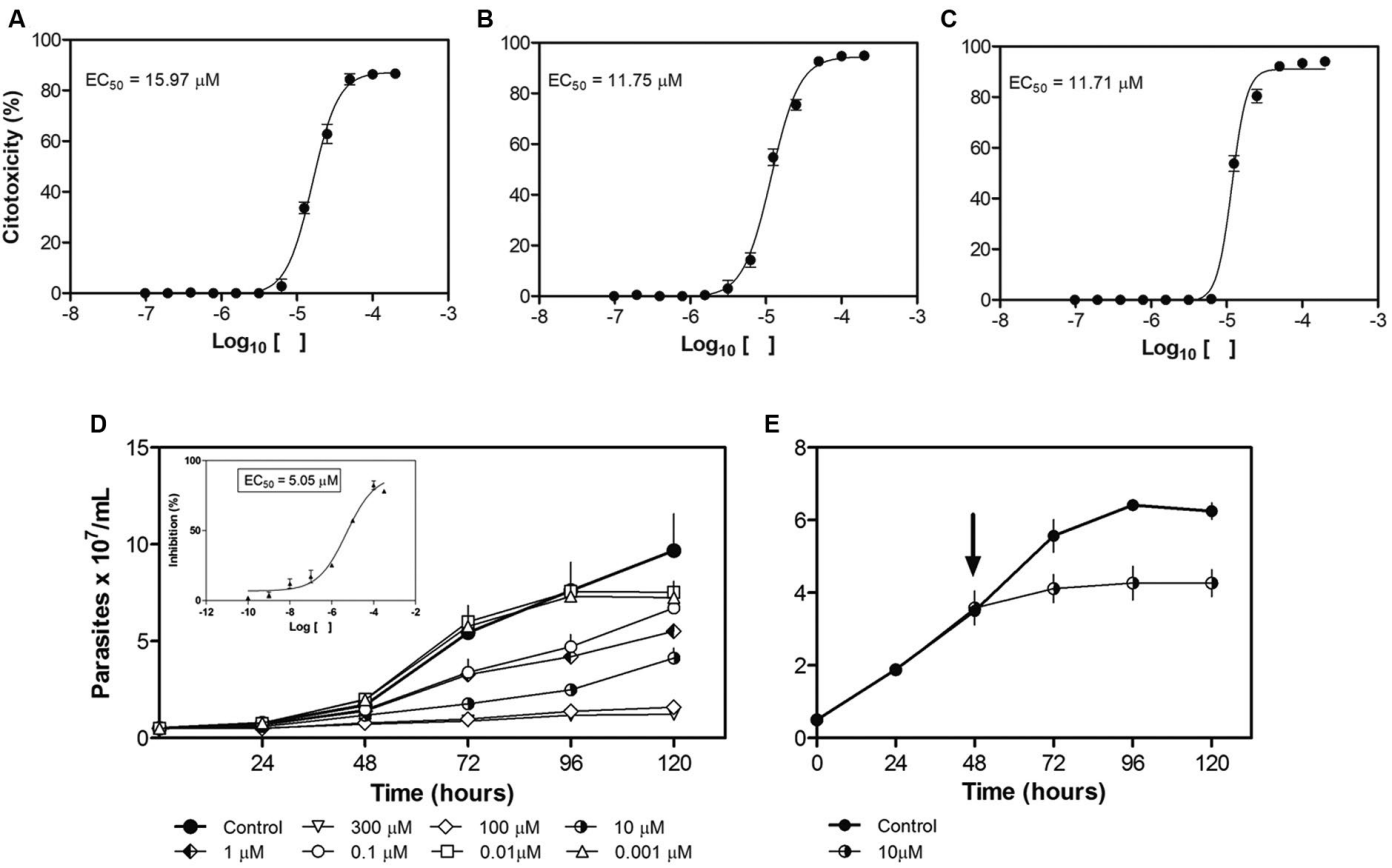
*Leishmania (Leishmania) amazonensis* promastigotes viability and proliferation. (A-C) Concentration-effect curves of ketoconazole on parasite viability by MTT after 24 h (A), 48 h (B), and 72 h (C) of treatment; (D) the concentration-response curves of ketoconazole on parasite proliferation after 24, 48, 72, 96 and 120 h of treatment. The smaller graph displays the respective EC_50_ value for 72 h. The cell density was obtained by direct counting in a Neubauer chamber of fixed cells. E. Assay on viable parasite proliferation in presence of 10 µM of ketoconazole. The arrow indicates the cultivation time in which the drug was added to the medium. The viable cell density was obtained by direct counting in a Neubauer chamber using Trypan blue stain. Data are represented as mean ± scanning electron microscopy (SEM).

*Ketoconazole inhibits growth parasite and interferes with parasite viability* - For evaluation of the antiproliferative effect of ketoconazole, promastigotes were incubated with different concentrations of the drug and the growth curve was followed daily up to 120 h. The drug inhibited the growth of the promastigotes in a concentration-dependent way, most notably after 72 h of treatment, with inhibition rates around 70% for 10 µM and 85% for 100-300 µM from 72 h onwards. (Fig. 1D: EC_50_ after 72 h = 5.0 µM). Once evidenced the antiproliferative effect of ketoconazole, a viability assay by MTT was carried out. Thus, promastigotes were incubated with several concentrations of ketoconazole for different times (24, 48 and 72 h) in order to assess whether the interference with viability could be preceding the effect on growth observed from 72 h onwards. Ketoconazole caused a concentration- and time-dependent inhibition on the *L. (L.) amazonensis* promastigote viability after 24, 48 and 72 h of incubation presenting EC_50_ values of 15.97, 11.75, and 11.71 μM, respectively (Fig. 1A-C). Despite the interference in parasite viability, at the drug concentration capable of inhibit 50% of the proliferation of parasites after 72 h of treatment (ketoconazole 5 µM), the parasites were viable. To confirm this result, another viability assay was performed: the Trypan Blue exclusion test. In the assay, the growth curve was followed up to 72 h and a single and higher ketoconazole concentration was used: 10 µM. The concentration used is close to the EC_50_ value of viability (72 h) and twice the EC_50_ of proliferation (72 h), which would make it possible to better observe the drug effect. For the other tests, this concentration was used. When parasites cultured for 48 h in absence of drug were exposed to ketoconazole (10 µM), a reduced number of viable parasites was observed, which remained constant over time, while the control parasites exhibited exponential growth (Fig. 1E). In other words, the ketoconazole interfered with parasite viability, but it is not killing the parasites over the time.

**Fig. 2:**
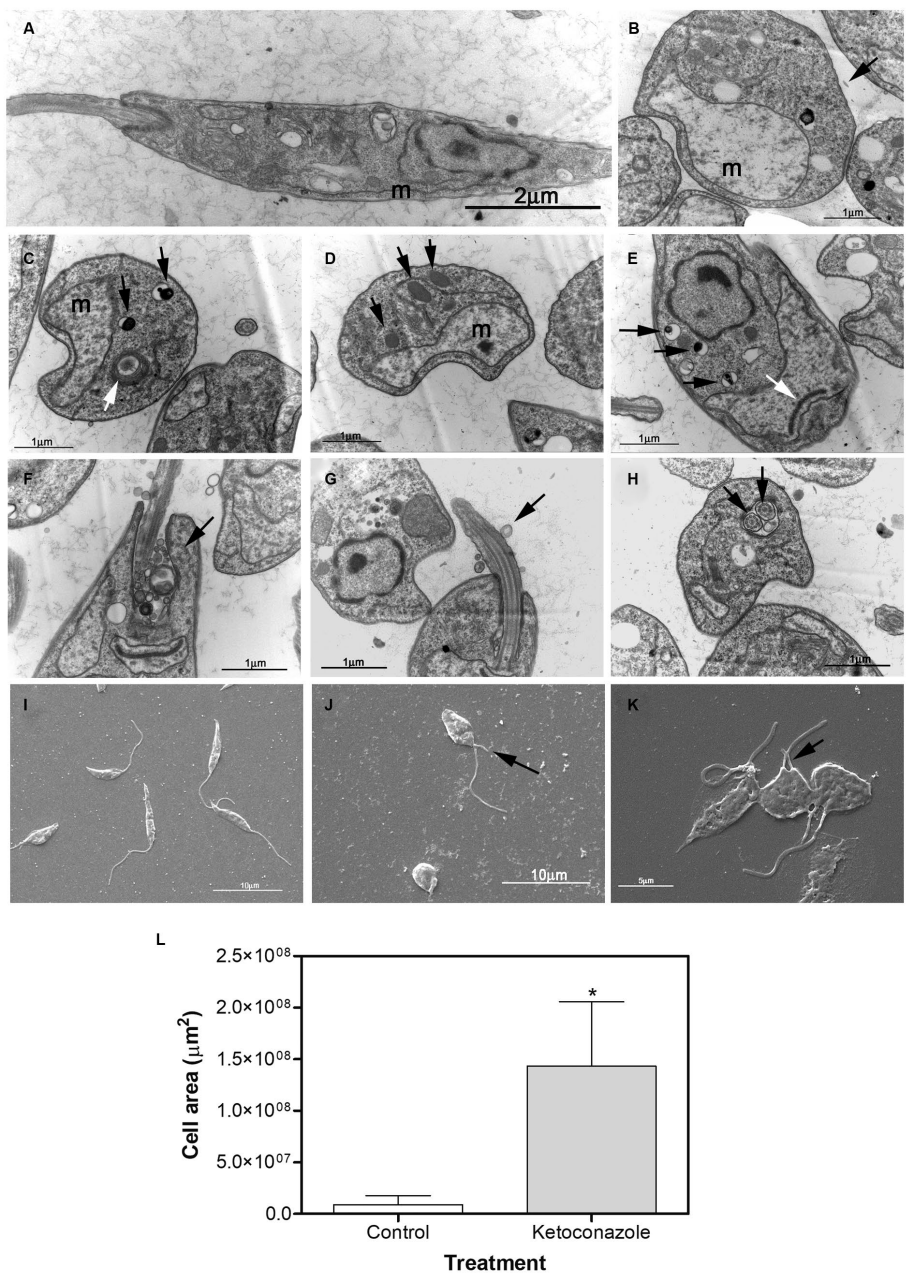
ultrastructural and morphological alterations observed in *Leishmania (Leishmania) amazonensis* promastigotes treated with ketoconazole. Transmission electron microscopy (TEM): (A) control parasite; (B-H) ketoconazole-treated parasites (10 µM); alterations are indicated by arrows: (B) mitochondrial swelling and intact cell membrane (black arrow); (C) vesicles associated to Golgi complex (white arrow) and acidocalcisomes (black arrows); (D) lipid droplets (black arrows); (E) acidocalcisomes (black arrows) and normal kinetoplast (white arrow); (F) intense activity of endo/exocitose at flagellar pocket (black arrow); (G) flagellar alterations (stumpy, detached membrane) (black arrow); (H) double flagella at flagellar pocket (black arrows). Scanning electron microscopy (SEM): (I) control parasite; (J-K) ketoconazole-treated parasites (10 µM) - Double flagella (black arrow). m = mitochondrion. (L) graph shows the quantification of mitochondrion area using the software ImageJ version 1.48. Data are expressed as the mean ± standard deviation (SD) and statistically significant difference compared to control was determined using ANOVA (p < 0.0001).

*Ketoconazole causes outstanding morphological and ultrastructural alterations* - In order to confirm the alterations previously observed and identify possible new damages, the ultrastructural analysis of the parasite was carried out. Promastigotes were incubated in absence (control) or presence of 10 µM of ketoconazole for 72 h. The control parasites exhibited normal cells with typical elongated and thin bodies, lengthened and single flagellum and mitochondrion (Fig. 2A, I). Ketoconazole-treated cells appeared rounded, swollen and with altered cell membrane morphology (Fig. 2 J-K) compared to non-treated ones, as revealed by scanning electron microscopy. Transmission electron microscopy revealed several morphological changes for treated-cells, such as significant mitochondrial swelling (Fig. 2B, L), vesicles associated to Golgi complex (Fig. 2C), augmented accumulation of acidocalcisomes (Fig. 2C, E) and of lipid droplets (Fig. 2D), intense activity of endo/exocytosis at flagellar pocket (Fig. 2F), flagellar alterations (stumpy and detached membrane) (Fig. 2G), and double flagella at flagellar pocket (Fig. 2H, J, K). However, normal kinetoplast (Fig. 2E) was observed.

**Fig. 3:**
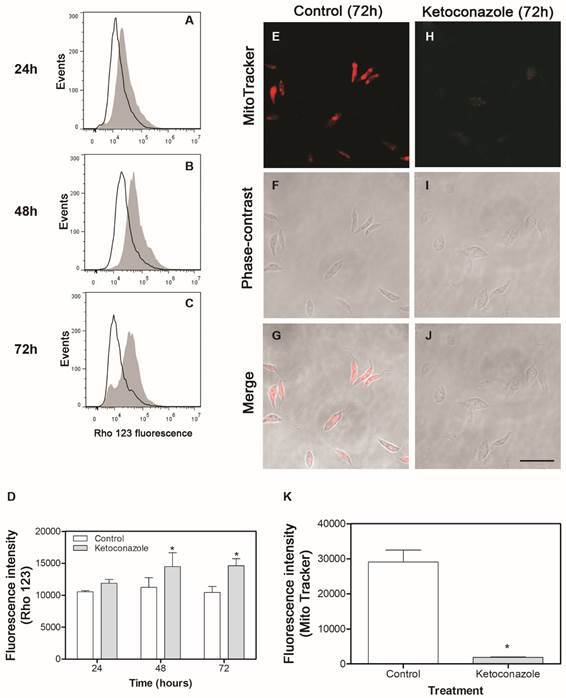
mitochondrial damage induced by ketoconazole on *Leishmania (Leishmania) amazonensis*. (A-C) flow cytometry histograms displaying changes in mitochondrial transmembrane potential (∆Ψ_m_) measured by Rhodamine 123 (Rho123) in different times of drug exposure (ketoconazole, 10 µM): 24 h (A), 48 h (B), and 72 h (C); (E-J) images obtained by confocal microscopy showing parasites cultured in absence (control, panels E, F and G) or presence of ketoconazole (panels H, I and J) for 72 h and submitted to staining with Mitotracker^R^. Bar: 10 µm. (D and K) the graphs show the quantification of fluorescence emitted by Rhodamine 123 (D) or Mitotracker^R^. (K) using the software ImageJ version 1.48. Data are expressed as the mean ± standard deviation (SD) and statistically significant difference compared to control was determined using ANOVA (p < 0.05).

*Ketoconazole interferes with mitochondrial activity and induces autophagic vacuoles formation* - In view of both the drug interference with the parasites viability and the significant mitochondrial swelling, the mitochondrial activities by Rhodamine 123 (Rho 123) and MitoTracker stains were evaluated. Furthermore, the autophagic vacuoles formation was assessed by MDC (monodansylcadaverine) labeling. Ketoconazole elevated mitochondrial activity, as suggested by the increased mitochondrial transmembrane potential (∆Ψ_m_) measured by Rho123 dye. A time-dependent increase in fluorescence was induced by the drug until 72 h of treatment (Fig. 3A-C). The fluorescence intensities of Rho 123 at each time (24, 48 and 72 h) are showed in the graph (Fig. 3D). Moreover, an intense labeling with MitoTracker^R^ for control parasites and an outstanding decrease in intensity for ketoconazole-treated promastigotes for 72 h (Fig. 3E-K) could be observed, which indicates loss of mitochondrial activity in these parasites.

**Fig. 4:**
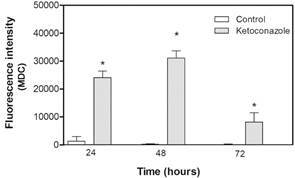
ketoconazole-induced autophagic vacuoles on *Leishmania (Leishmania) amazonensis* promastigotes. Parasites were incubated with 10 µM ketoconazole for different times (24, 48, and 72 h) and then submitted to MDC staining. The fluorescence intensities were determined by the software ImageJ version 1.48. The graph shows the quantification of fluorescence. Data are expressed as the mean ± standard deviation (SD) and statistically significant difference compared to control was determined using ANOVA (p < 0.001).

Autophagic vacuoles were intensely labeled at 24 and 48 h after drug incubation. However, such marking was shown to be reduced after 72 h (Fig. 4). There was statistically significant difference between ketoconazole-treated samples (24, 48, and 72h) compared to control non-treated, with p < 0.001.

**Fig. 5:**
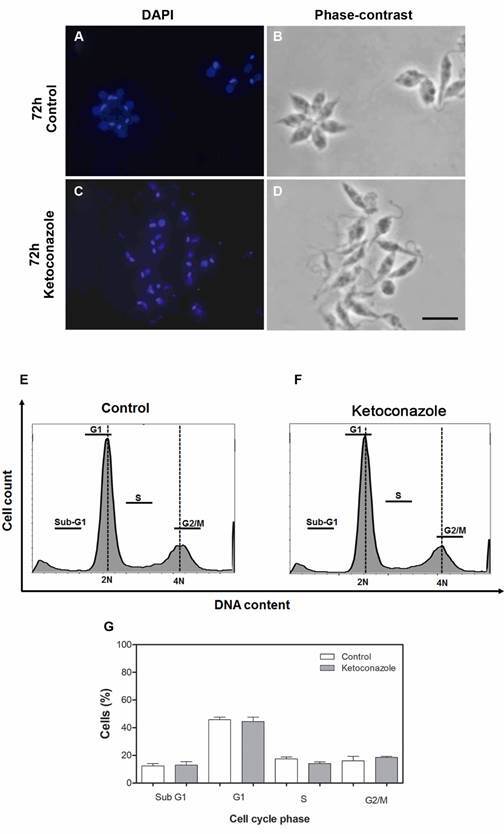
cell cycle assay. (A-D) images obtained by confocal microscopy to nucleus, kinetoplast and flagellum counting showing parasites cultured in absence (control, panels A-B) or presence of ketoconazole (10 µM, 72 h) (panels C-D) and submitted to staining with DAPI. Bar: 10 µm. (E-F) typical DNA content frequency histograms representing *Leishmania (Leishmania) amazonensis* promastigotes incubated with: (E) medium, control or (F) ketoconazole (10 µM; 72 h). The cells were stained with propidium iodide (PI) and fluorescence of the PI-stained cells was measured. Cell cycle analysis provides the estimate of percentage of cells in Sub-G1, G1, S and G2/M phases of the cycle. G. The graph shows the quantification of fluorescence by the software ImageJ version 1.48. Data are expressed as the mean ± standard deviation (SD) and statistically significant difference compared to control was not observed using ANOVA (p < 0.05).

*Ketoconazole does not alter the parasite cell cycle* - Due to growth inhibition and presence of double flagella and other flagellar alterations observed by electron microscopy analysis, the interference of the drug with the parasite cell cycle was investigated (treated parasites incubated with 10 µM of ketoconazole for 72 h). The interference of the drug with the cell cycle of *L. (L.) amazonensis* promastigotes, which was determined by counting of nucleus, kinetoplast and flagellum, did not reveal differences (data not shown) between treated and control parasites. The cell cycle analysis carried out by flow cytometry showed that the treatment did not cause alterations in a specific phase of cell cycle. In other words, the treatment does not interfere with the parasite cycle under the conditions tested (Fig. 5).

**Fig. 6:**
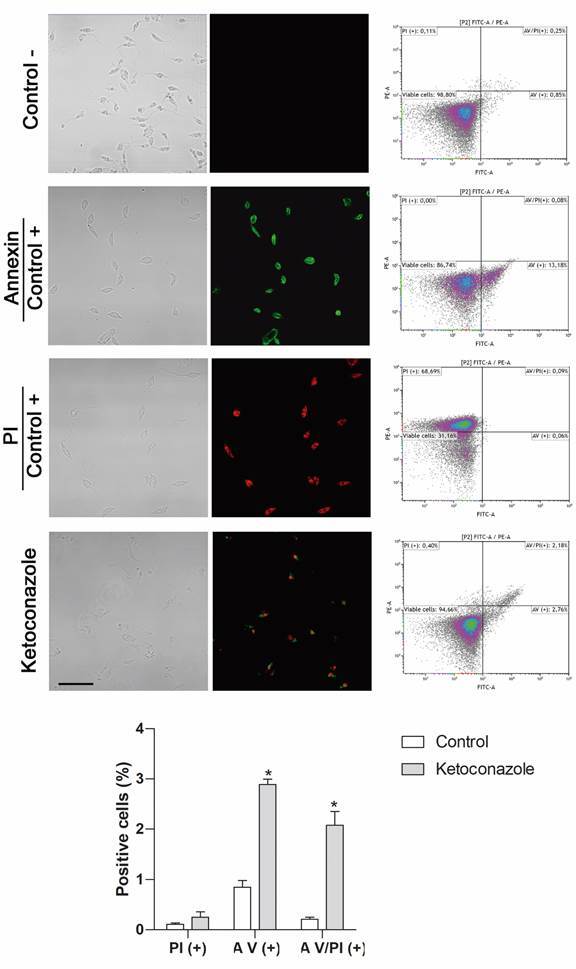
cell death assay. Images obtained by confocal microscopy showing parasites cultured in presence of medium (negative control), formaldehyde 4% (positive control) or ketoconazole (10 μM, 72 h) and submitted to staining with propidium iodide (PI) and/or Annexin V. Bar: 20 µm. Respective representative dot plots for Annexin V-FITC (A V) and/ or PI staining in *Leishmania (Leishmania) amazonensis* promastigotes. Lower left quadrant, viable cells (negative for both A V and PI); lower right quadrant, initial apoptotic cells (positive for A V and negative for PI); upper right quadrant, late apoptotic cells (positive for both A V and PI) and upper left quadrant, necrotic cells (positive for PI and negative for A V). The graph indicates the percentage of positive cells for PI, A V and both (A V/PI) for ketoconazole-treated parasites in relation to negative control. *: statistically significant difference compared to negative control with p < 0.001.

*Ketoconazole tends to induce cell death by apoptosis* - Given that ketoconazole causes several effects on *L. (L.) amazonensis* promastigotes (viability interference, mitochondrial swelling, increase in the amount of acidocalcisomes, induction of autophagic vacuoles), but does not alter the cell cycle, it was evaluated the type of cellular death that was occurring. The dot plot analysis of the assays (PI/Annexin V labeling and flow cytometry; treated parasites incubated with 10 µM of ketoconazole for 72 h; Fig. 6) showed that the distribution of viable, necrotic, and apoptotic cells after treatment with ketoconazole for 72 h was similar to that observed for untreated parasites (negative control), tending to cause apoptosis (4.94% *versus* 1.1%, statistically significant difference with p < 0.001 for ketoconazole and control, respectively), under the conditions tested.

**Fig. 7:**
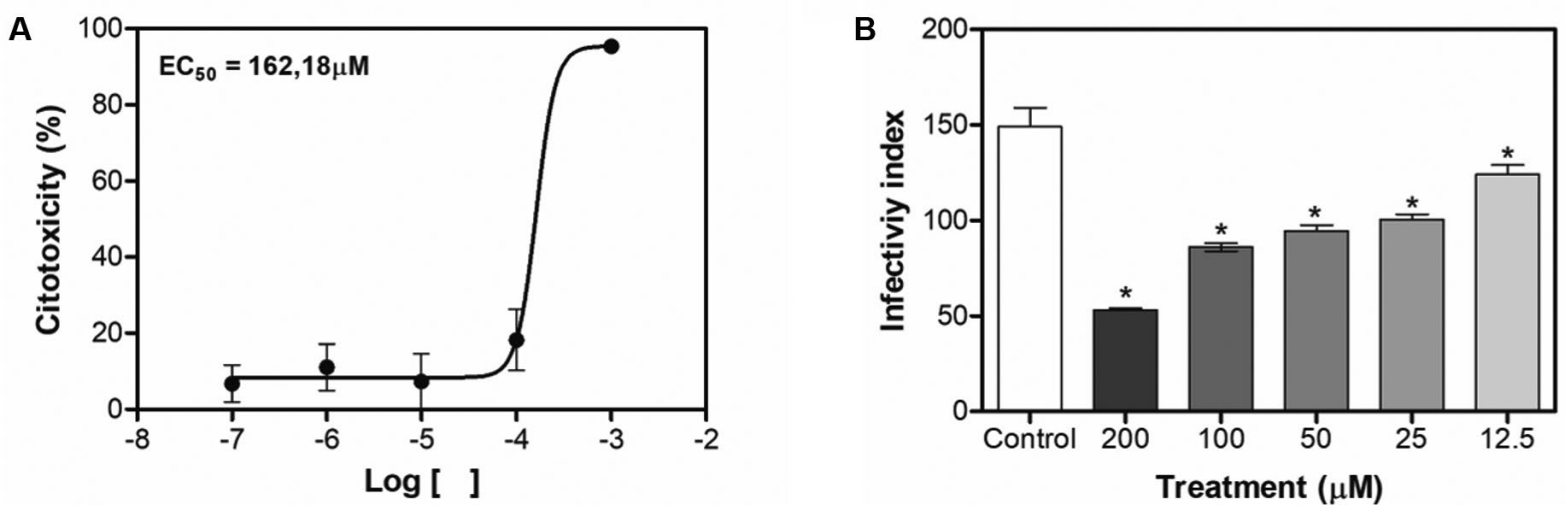
viability of murine macrophages cell line RAW264.7 and infective capacity of amastigotes (infectivity). Concentration-effect curve of ketoconazole on macrophages by MTT after 48 h of treatment (A). Infected macrophages were treated with ketoconazole (200-12.5 µM) for 48 h. The infectivity index was determined by multiplying the percentage of macrophages that had phagocytosed at least one parasite by the parasite average per infected macrophage (100 cells were examined) and are shown in the graph (B). Data are expressed as mean ± scanning electron microscopy (SEM). Statistically significant difference compared to negative control with p < 0.05.

*Ketoconazole is not toxic for host cells and interferes with infective capacity of amastigotes* - In view of the effects induced by ketoconazole on the promastigote parasites, intracellular amastigotes, the clinically relevant form of the parasite, were treated with different concentrations of ketoconazole for 48 h in attempt to evaluate eventual interference of drug on infective capacity. For this purpose, a viability assay was previously carried out for the host cell (murine macrophage cell line RAW264.7). Concentration-dependent inhibition on macrophages viability was observed, with EC_50_ of 162.18 µM at 48 h. At infectivity condition, the drug interfered with infective capacity of amastigotes. After 48 h of treatment, similar drug concentrations: 11.75 µM (EC_50_ for promastigote - 48 h; Fig. 1B) and 12.5 µM (lowest tested concentration in the infectivity assay; Fig. 7A) inhibited 50% and 17% of promastigotes and intracellular amastigotes (infectivity), respectively. It is important to note that these concentrations were not toxic to the host cell, as the EC_50_ for macrophages was about 13-14 times higher.

## DISCUSSION

Considering the importance of ergosterol for the biology and pathogenesis of the *Leishmania* parasite, herein, we certified the antiproliferative/viability effects of ketoconazole against *L. (L.) amazonensis* both promastigotes^31^ and intracellular amastigotes^32^ in another parasite strain: IFLA/BR/67/PH8. Furthermore, we show in more detail the biological effects of the inhibitor, aiming at understanding the drug mechanism of action.

Regarding ultrastructure, our results revealed rounded up shape, mitochondrial swelling, augmented accumulation of lipid droplets and acidocalcisomes, presence of multivesicular bodies and frequent truncated and/or double flagella. These data are similar to those previously obtained for *L. amazonensis* promastigotes treated with ketoconazole^31^ ^,^ ^33^ ^,^ ^34^ and suggest mitochondrial damage.

When parasites (*Trypanosoma cruzi*, *Leishmania* spp, *Toxoplasma gondii*) are treated with inhibitors of important enzymes of the ergosterol biosynthesis pathway, the mitochondrion is the organelle primarily affected.^35^ In our study, the ketoconazole-treated parasites presented hyperpolarisation of the mitochondrial membrane potential, time-dependent up to 72 h. Mitochondrial membrane potential variations could be the result of diverse events: inhibition of electron transport chain (decrease); blockage of ATP synthase (increase); stimulation of uncoupling proteins (decrease); or permeabilisation of the inner membrane (decrease).^36^ Macedo et al.^37^ demonstrated that *L. (L.) amazonensis* promastigotes treated with itraconazole and posaconazole for 48 h presented collapse of the mitochondrial membrane potential associated with intense mitochondrial swelling, disorganisation and rupture of mitochondrial membranes. Another study showed that *T. cruzi* treated with ketoconazole (EC_50_ = 32 µM for 72 h) presented a gradual increase in Rho 123 time-dependent fluorescence and a confocal microscopy of parasites confirmed an intense proliferation of the inner mitochondrial membrane. This organelle became highly branched and compact and elevated levels of Rho 123 were accumulated within the cells.^38^ In many cases, a transient hyperpolarisation occurs before mitochondrial depolarisation, as if it were an attempt of the cells to avoid death. This effect can be seen in great part of the heat-shocked *Leishmania* promastigotes.^39^

The mitochondrial membrane is hyperpolarised does not mean that the organelle is functioning normally. Our results showed that MitoTracker^R^ labeling, a probe which passively diffuses across the plasma membrane and accumulates in active mitochondria, revealed an outstanding intensity decrease in ketoconazole-treated promastigotes, indicating loss of mitochondrial activity in these promastigotes. This result was also confirmed by the MTT viability assay, which measures the activity of mitochondrial enzymes, and by the proliferation curve using Trypan blue, which takes into account only viable cells. Mitochondrion is essential for generating the necessary energy for the survival and proliferation of eukaryotic cells.^40^ ^)^ In this way, malfunctioning mitochondria could impair the ATP synthesis and inorganic phosphate would accumulate in acidocalcisomes, which explains the increase in the amount of this organelle 72 h after ketoconazole treatment. Acidocalcisomes are organelles of acidic nature and high electron density important due to their storage of polyphosphates, calcium, magnesium, and other elements.^41^ The augmented number of this organelle was already reported for ketoconazole and *L. amazonensis* (MHOM/Josefa/75/Br strain).^42^

The treatment of *L. amazonensis* with ketoconazole stressed the cells, especially early after the treatment (24 and 48 h), leading to the appearance of autophagic vacuoles. Itraconazole and posaconazole induced appearance of autophagosome-like structures in *L. (L.) amazonensis* ^37^ and Rodrigues et al.^43^ described the effect of autophagy on *L. amazonensis* (MHOM/BR/75/Josefa strain) treated with 22,26-azasterol. Thus, the confirmed presence of autophagic vacuoles might represent an adaptive response of the parasite to treatment (stress condition). Initially, parasites would resort to autophagic vacuoles in an attempt to remodel/remove abnormal cellular constituents, degrading damaged structures.^44^ Over time, directing energy to the parasite single mitochondrion may be more important than repairing cell damage. This would explain the fact that at 72 h there was less labeling for autophagic vacuoles and higher mitochondrial hyperpolarisation occurred. Autophagy is involved in turnover and recycling by removal of damaged cellular components, regulating homeostasis during crucial processes such as cell growth and differentiation and plays fundamental role in mitochondrial functionality.^44^ ^,^ ^45^

Results of the growth curve and frequent ultrastructural alterations in flagellum (truncated and/or double flagella), despite the intact kinetoplast, suggest that ketoconazole interferes with the *L. amazonensis* cell cycle. Therefore, the cell cycle of the parasites was evaluated both by nucleus, kinetoplast, and flagellum (n-k-f) counts and by flow cytometry. Different from some data in the literature involving ketoconazole acting on other cell types^46^ ^,^ ^47^ and azole compounds on *L. amazonensis*,^37^ our results of the n-k-f score did not reveal differences between the control and ketoconazole-treated parasites. In addition, the cell cycle assay showed that the treatment was not able to alter the cycle phases (Sub G1, G1, S, and G2/M) in relation to control parasites suggesting that ketoconazole does not interfere with parasite replication under the tested conditions. When *L. (L.) amazonensis* was treated with itraconazole and posaconazole, some cells presented more than two flagella and significant alterations in kinetoplast were observed by ultrastructural analysis; however, the possible alteration in the cell cycle was not evaluated.^37^

Ketoconazole has been related to apoptosis in several normal and tumoral cells.^48^ ^,^ ^49^ ^,^ ^50^ ^,^ ^51^ Haegler et al.^52^ showed that ketoconazole and posaconazole presented hepatocellular toxicity, impairing the ∆Ψ_m_, the function of enzyme complexes of electron transport chain, accumulating mitochondrial superoxide and inducing apoptosis. Possibly, mitochondrial dysfunction could be associated with hepatotoxicity caused by those azole compounds. Our data revealed that treatment of the parasites with ketoconazole, under the conditions previously described, showed a trend towards apoptosis, although there was a slight positive result for PI. Furthermore, few autophagic vacuoles were observed after treatment. Other researchers reported that treatment of *T. cruzi* with ketoconazole (EC_50_/72 h) resulted in neither necrosis nor apoptosis. However, high concentrations of the drug (EC100/24h) resulted in fast cell death due to necrosis.^38^

SBIs effects on extracellular *L. amazonensis* parasites (promastigotes) appear to be more gradual than that observed with the intracellular form (amastigote);^31^ thus, SBIs effects profile allows a more detailed study of the events in the promastigote form. In view of the effects provoked by ketoconazole on the *L. (L.) amazonensis* promastigote, the interference of drug on infectivity capacity of amastigotes, the clinically relevant form of the parasite, was evaluated and evidenced a susceptibility to treatment. Taken together, the infectivity results added to the data of mitochondrial damage, increase in the acidocalcisome and autophagic vacuoles amounts may be evidence that the susceptibility observed *in vitro* is related to mitochondrial dysfunction provoked by ketoconazole. However, future studies should be carried out on amastigotes to confirm this hypothesis.

**Fig. 8:**
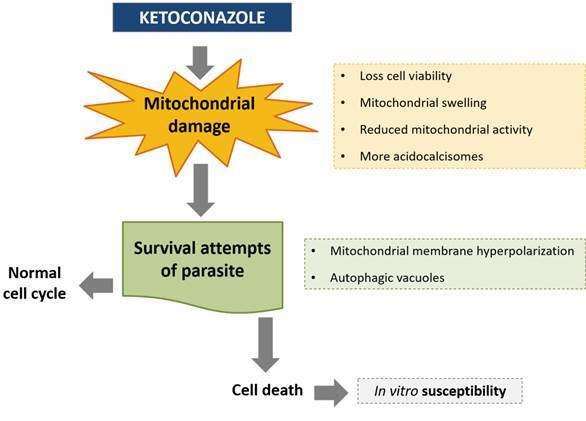
proposed mode action of ketoconazole on Leishmania (Leishmania) amazonensis. Parasites resort to autophagic vacuoles and mitochondrion hyperpolarisation in attempt to survive to damages mainly in the mitochondrion. The efforts to maintain the cell energy are not sufficient since the decrease of cell and mitochondria viability and increase of acidocalcisomes. These damages do not interfere with the parasite cell cycle, but they lead the parasites to death, making them susceptible to in vitro treatment conditions.

To our knowledge, this study demonstrates the effects of ketoconazole on mitochondrion functioning, autophagic compartments, cell cycle and death of *L. (L.) amazonensis* promastigotes for the first time. We suggest that some damages are occurring in the parasite, mainly in the mitochondrion. In an attempt to remodel and/or destroy eventual damaged structures, parasites hyperpolarise mitochondrion and resort to autophagic vacuoles. However, this survival strategy adopted by the cell to direct efforts to maintain the cell energy is not sufficient because of the decrease in cell and mitochondria viabilities and increase in acidocalcisomes. Although these damages do not reflect directly in the parasite cell cycle, they lead the parasites to death, making them susceptible to *in vitro* treatment (Fig. 8).
